# Associations of maternal folic acid supplementation and folate concentrations during pregnancy with foetal and child head growth: the Generation R Study

**DOI:** 10.1007/s00394-015-1058-z

**Published:** 2015-10-26

**Authors:** Jolien Steenweg-de Graaff, Sabine J. Roza, Alette N. Walstra, Hanan El Marroun, Eric A. P. Steegers, Vincent W. V. Jaddoe, Albert Hofman, Frank C. Verhulst, Henning Tiemeier, Tonya White

**Affiliations:** 1 The Generation R Study Group, Erasmus Medical Centre, Rotterdam, The Netherlands; 2 Department of Child and Adolescent Psychiatry/Psychology, Erasmus MC-Sophia, P.O. Box 2060, 3000 CB Rotterdam, The Netherlands; 3 Department of Psychiatry, Erasmus Medical Centre, Rotterdam, The Netherlands; 4 Department of Psychiatry, Leiden University Medical Centre, Leiden, The Netherlands; 5 Department of Obstetrics and Gynaecology, Erasmus Medical Centre, Rotterdam, The Netherlands; 6 Department of Paediatrics, Erasmus Medical Centre, Rotterdam, The Netherlands; 7 Department of Epidemiology, Erasmus Medical Centre, Rotterdam, The Netherlands; 8 Department of Radiology, Erasmus Medical Centre, Rotterdam, The Netherlands

**Keywords:** Folate, Folic acid supplementation, Pregnancy, Foetal neurodevelopment, Head growth, Head size

## Abstract

**Purpose:**

Folic acid supplementation during pregnancy has been associated with a reduced risk of common neurodevelopmental delays in the offspring. However, it is unclear whether low folate status has effects on the developing brain. We evaluated the associations of maternal folic acid supplementation and folate concentrations during pregnancy with repeatedly measured prenatal and postnatal head circumference in the offspring.

**Methods:**

Within a population-based prospective cohort, we measured maternal plasma folate concentrations at approximately 13 weeks of gestation (90 % range 10.5–17.2) and assessed folic acid supplementation by questionnaire (2001–2005). Up to 11 repeated measures of head circumference were obtained during foetal life (20 and 30 weeks of gestation) and childhood (between birth and age 6 years) in 5866 children (2002–2012).

**Results:**

In unadjusted models, foetal head growth was 0.006 SD (95 % CI 0.003; 0.009, *P* < 0.001) faster per week per 1-SD higher maternal folate concentration. After adjustment for confounders, this association was attenuated to 0.004 SD per week (95 % CI 0.000; 0.007, *P* = 0.02; estimated absolute difference at birth of 2.7 mm). The association was independent of overall foetal growth. No associations were found between maternal folate concentrations and child postnatal head growth. Preconceptional start of folic acid supplementation was associated with larger prenatal head *size*, but not with prenatal or postnatal head *growth*.

**Conclusions:**

Our results suggest an independent, modest association between maternal folate concentrations in early pregnancy and foetal head growth. More research is needed to identify whether specific brain regions are affected and whether effects of folate on foetal head growth influence children’s long-term functioning.

**Electronic supplementary material:**

The online version of this article (doi:10.1007/s00394-015-1058-z) contains supplementary material, which is available to authorized users.

## Introduction

Supplementation with folic acid during embryonic development reduces the risk of neural tube defects in infants [1, 2]. This protective effect has led to recommendations for daily supplementation with folic acid starting preconceptionally in women planning to become pregnant. Additionally, some countries have adopted fortification of flour with folic acid [3].

Emerging evidence from observational research has shown that maternal folic acid supplementation during pregnancy is associated with reduced risk of several other neurodevelopmental disorders in offspring, including language delays [4], autism spectrum disorders [5, 6] and problem behaviour [7]. However, the underlying neurobiology of low folate and these neurodevelopmental problems remains unclear. Folic acid supplement use is strongly related to higher socioeconomic status and is a marker of good health literacy [8, 9], which increases the susceptibility for confounding.

To further support a potential relationship between folic acid supplement use and neurodevelopmental disorders, nutritional biomarkers and biological intermediates can be examined. Blood folate concentration serves as a key nutritional biomarker. This can be measured in maternal blood and can serve as the endpoint of the determinants of folate status, i.e. after physiological absorption of folic acid supplements and folates from natural (or fortified) foods [10]. Unlike folic acid supplementation, folate concentrations are unaffected by recall bias, thus serving as a better proxy for actual folate status.

Biological intermediates include structural alterations in the brain throughout pregnancy and early postnatal development. Head circumference (HC) can serve as a non-invasive and inexpensive proxy for foetal and early postnatal brain growth and development. HC is closely related to brain volume, particularly in the period before the skull is fully developed [11, 12]. Several neurodevelopmental disorders are associated with subtle changes in head growth. Children with autism for instance have normal to slightly smaller HC at birth, followed by accelerated head growth in the first year of life when compared to non-autistic children [13].

Folate is involved in a number of essential processes of growth and development, such as DNA synthesis and gene expression. Consequently, folate requirements are increased in periods of rapid growth, such as pregnancy. Low maternal folate concentrations directly limit the availability of folate to the foetal cells, which results in impairment of cell division and potentially impairs growth [14].

Earlier research provides inconsistent evidence for an association between maternal folate concentrations and HC at birth, and no evidence for an association between maternal folic acid supplementation and HC at birth [15]. In a previous report of the current cohort, maternal folic acid supplementation was associated with head size in prenatal life, but foetal head *growth* was not considered as a pregnancy outcome [16]. Whether prenatal folate concentrations or maternal folic acid supplementation during pregnancy has sustained or additive effects on postnatal head growth is unknown.

We used the Generation R cohort to evaluate the associations of both maternal folic acid supplementation and folate concentrations during pregnancy with repeatedly measured offspring prenatal and postnatal head size. We hypothesised that folic acid supplementation as well as higher folate concentrations is associated with increased head growth in the offspring.

## Methods

### Study population

The subjects were participants in the Generation R Study, a population-based cohort from early foetal life onwards in Rotterdam, the Netherlands [17]. The study was conducted in accordance with the guidelines proposed in the World Medical Association Declaration of Helsinki and was approved by the local medical ethics committee. Written consent was obtained from all participants.

In total, 8879 women were enrolled in the Generation R Study during pregnancy. Out of 8782 women with a singleton pregnancy, 6993 women (80 %) enrolled in the study before 18 weeks of gestation. We measured plasma folate concentration in early pregnancy in 5965 (85 %) of these women. We excluded 37 women since their measure of folate was above the assay range, resulting in valid folate concentrations at baseline for 5928 mothers. Twenty children died during the neonatal period and one was lost to follow-up before birth; these were excluded from this study. In total, at least one measure of foetal or child HC was obtained in 5866 children.

### Maternal folic acid supplement use

Before 18 weeks of gestation (median 13.2 weeks; 90 % range 10.5–17.2 weeks), pregnant women were asked by questionnaire whether they used folic acid supplements or multivitamin preparations and when they started supplementation. In the Netherlands, both folic acid supplements and multivitamin preparations for pregnant women contain 400–500 µg folic acid; there is no mandatory food fortification with folic acid. In line with previous publications, we categorised folic acid supplement use in four groups: (1) preconceptional start (as recommended [18]) (*n* = 2153), (2) start within the first 10 weeks of pregnancy (*n* = 1602), (3) start after the first 10 weeks of pregnancy (*n* = 753) and (4) no use (*n* = 1358) [19]. To distinguish between effects of folic acid and multiple micronutrients, we separated preconceptional start into ‘folic acid only use’ (*n* = 1396) and ‘multivitamin use’ (*n* = 757) in additional analyses.

### Maternal folate concentrations

In early pregnancy (median 13.2 weeks; 90 % range 10.5–17.2 weeks), venous blood samples were drawn, centrifuged and stored at −80 °C, as previously described [20]. After thawing, folate concentrations were analysed in 2008, using an immunoelectrochemoluminence assay. The between-run coefficients of variation for plasma folate varied between 1.5 and 8.9 %, with an analytic range of 1.8–45.3 nmol/L (0.8–20.0 ng/mL).

Folate concentration was analysed:

- as a continuous variable (standard deviation scores (SDS)) and
- as a dichotomous variable, categorised into ‘folate-deficient’ (<7 nmol/L = 3.1 ng/mL, 8.4 %) [10] or ‘normal folate concentration’.

Additional analyses were performed using a different cut-off (based on normative concentrations determined by the Erasmus Medical Centre laboratory: cut-off <8 nmol/L (3.5 ng/mL); 13.8 %).

### Foetal and child head circumference

Foetal ultrasound measurements were performed at prenatal visits during each trimester of pregnancy [17, 21]. First trimester ultrasounds were primarily used for pregnancy dating [21, 22]. The intra- and interobserver reliabilities of foetal biometry in early pregnancy were excellent (all intra-class correlation coefficients greater than 0.98) [23].

HC was subsequently measured at birth and in up to seven visits at child healthcare centres at the ages of 0–2, 2, 3, 4, 5–10, 10–13 and 13–17 months. Gender of the child and gestational age at birth were extracted from medical records. HC was again measured at the Generation R research centre (mean age of 6.2 years).

Gender and gestational age-adjusted SD scores were constructed for all measures of HC based on growth reference curves [21, 24]. This approach enables linear analyses of (nonlinear) growth patterns [25].

### Covariates

Several maternal and child characteristics were selected as possible confounding variables, based on previous studies of maternal folate or foetal development [15, 26]. These were maternal age and body mass index (BMI) at enrolment, national origin, educational level, smoking and alcohol consumption during pregnancy, psychopathology in mid-pregnancy (highest prevalence for anxious and depressive symptoms), parity, family income, foetal/child gender and gestational age at time of the HC measurements. Assessment of these covariates has been described in detail previously [19, 26, 27].

### Statistical analyses

Folic acid supplementation and folate concentration were used as the independent variables in the analyses. In the analyses of folic acid supplementation, the group of mothers who did not use folic acid supplements comprised the reference group. In the analyses of folate deficiency, the group of mothers with normal folate concentrations comprised the reference group. We additionally used linear regression models to evaluate the associations between maternal folate concentrations with offspring head *size* at all separate time points of HC measurement.

To test the associations with offspring head *growth*, we performed longitudinal analyses using unbalanced repeated measures analysis (mixed models). These analyses were performed separately for prenatal and postnatal head growth, since growth patterns as well as measurement methods differ between these developmental stages. Using maternal folate concentration as the independent variable, the model can be written as follows:

$${\text{SD}}\,{\text{score}}\,{\text{of}}\,{\text{HC}} = \beta_{0} + \beta_{1} \times {\text{gawks}} + \beta_{2} \times {\text{folate}} + \beta_{3} \times {\text{gawks}} \times {\text{folate}} + \varepsilon$$

(*β*_0_ = intercept; gawks = gestational age in weeks; *ε* includes additional covariates for the adjusted model and error; similarly, a formula for folic acid supplementation as the independent variable can be defined). The term ‘*β*_2_ × folate’ tests the difference in intercept for each 1-SD higher folate concentration or comparing the folate-deficient group to the reference group. Gestational age was centred at 20 weeks of gestation in the prenatal models.

Coefficient β_3_ reflects the slope (interaction of gestational age and folate) and represents the average decline or increase in head growth per gestational week for each 1-SD higher folate concentration or in children of folate-deficient mothers versus the reference group. Thus, the weekly change in the SD score is multiplied by the average number of weeks of pregnancy to obtain the expected difference at birth. A random-effects model was applied for the intercept and gestational age to account for within- and between-individual variation. All other covariates were fitted as fixed effects. Longitudinal analyses were additionally adjusted for the effects of maternal ethnicity, smoking and educational level on head growth per week [28–31]. We also added a quadratic term of the continuous folate variable to test for nonlinear associations. Additionally, to further explore nonlinearity and potential effects of very high folate status, we (1) divided maternal folate concentration into quintiles and (2) compared the highest 10 % (folate > 30.2 nmol/L, *n* = 588) and 5 % (folate > 33.8 nmol/L, *n* = 292) of the distribution of folate concentrations with the remaining 90 and 95 %, respectively, in secondary analyses.

Basic models were adjusted for gestational age at venipuncture, and gender and age of the foetus/child at assessment, if applicable. Subsequent models were additionally adjusted for the covariates mentioned earlier. Further, in the event of a significant association, we additionally adjusted for estimated foetal weight (EFW) to test whether maternal folic acid supplementation or folate status specifically affects offspring HC size/growth independent of overall growth. Finally, we explored potential effect modification by child gender in the fully adjusted models by including the interaction term between maternal folic acid use/folate concentration and child gender.

Missing values of covariates were imputed using the Markov Chain Monte Carlo multiple imputation technique with predictive mean matching for continuous variables (0.1–21.4 % missing data). Five imputed datasets were generated. Subsequently, analyses were performed separately on each completed dataset and thereafter combined to one pooled estimate.

## Results

Descriptive characteristics of the participating mothers and their children are presented in Table 1.

**Table 1:** Subject characteristics by maternal folate deficiency^a^ in early pregnancy^b^

	Folate-deficient *n* = 493 (8.4 %)	Normal folate concentrations *n* = 5373 (91.6 %)	*P* ^c^
Child characteristics			
Gender (% girl)	49.5	47.8	0.50
Gestational age at birth (weeks)^d,e^	39.7 ± 2.2	39.9 ± 1.7	<0.01
Maternal characteristics			
Age at enrolment (years)	27.0 ± 5.5	30.0 ± 4.9	<0.001
Gestational age at enrolment (weeks)^f^	14.3 ± 2.2	13.5 ± 2.0	<0.001
Ethnicity (%)			
Dutch	28.9	53.9	
Other Western	7.1	9.0	
Turkish or Moroccan	24.8	13.7	<0.001
Surinamese or Antillean	21.1	10.6	
Other non-Western	18.1	12.8	
Education (%)			
Higher	14.5	45.8	
Secondary	62.7	44.5	<0.001
Primary	22.8	9.7	
Family income (%)			
>2000 €/month	23.9	60.7	
1200–2000 €/month	25.5	19.6	<0.001
<1200 €/month	50.6	19.7	
BMI at intake (kg/m^2^)	25.5 ± 5.0	24.4 ± 4.3	<0.001
Smoking during pregnancy (%)			
Never	62.3	75.4	
Until pregnancy was known	6.0	8.5	<0.001
Continued throughout pregnancy	31.7	16.1	
Alcohol consumption during pregnancy (%)			
Never	62.2	43.7	
Until pregnancy was known	13.5	14.1	<0.001
Continued throughout pregnancy	24.3	42.2	
Parity, primiparae (%)	42.8	58.4	<0.001
Psychopathology in mid-pregnancy^g^	0.42 ± 0.47	0.28 ± 0.37	<0.001
Folic acid supplement use during pregnancy (%)			
No use	72.5	18.6	
Start > 10 weeks pregnancy	11.0	13.0	<0.001
Start ≤ 10 weeks pregnancy	10.0	28.9	
Preconceptional start	6.5	39.5	

^a^Folate deficiency was defined as a folate concentration <7 nmol/L

^b^Descriptives on imputed data

^c^Derived from linear regression analysis for continuous variables or logistic regression analysis for categorical variables on imputed data

^d^Means ± SD (all such values)

^e^Median (95 % range): deficient group: 39.9 (34.4–42.3); nondeficient group: 40.1 (35.8–42.3)

^f^Median (95 % range): deficient group: 14.4 (9.8–17.8); nondeficient group: 13.2 (9.6–17.5)

^g^Median (95 % range): deficient group: 0.25 (0.0–1.92); nondeficient group: 0.15 (0.0–1.39)

### Maternal folic acid supplementation and offspring head circumference

Table 2 shows the analyses of the association of maternal folic acid supplementation with offspring head size and growth. In the basic model, foetuses of mothers who started folic acid supplementation before conception or within 10 weeks of pregnancy had a slightly larger head *size* at 20 weeks of gestation compared with children of mothers who did not use supplements during pregnancy. After adjustment for confounders, this association was only observed in foetuses of mothers who started supplementation before conception, similar to the previous report [16]. Maternal ethnic background, income, BMI at enrolment and smoking during pregnancy were the main confounders. Additional adjustment for EFW slightly attenuated the effect estimate, but the association remained statistically significant (*B* = 0.112, 95 % CI 0.028; 0.196, *P* < 0.01).

**Table 2:** Maternal folic acid supplementation during pregnancy and offspring head size at 20 weeks of gestation, prenatal head growth and postnatal head growth^a^

	Head circumference size (SDS)					
	Basic^b^			Adjusted for covariates^c^		
	SDS^d^	B^e^ (95 % CI)	*P*	SDS^d^	B^e^ (95 % CI)	*P*
Head size (intercept (SDS) at 20 weeks gestation) and folic acid supplementation (*n* = 5832 (99.4 %), 11,323 observations)						
No use^f^	**−**0.163	Reference		**−**0.003	Reference	
Start > 10 weeks	**−**0.135	0.028 (**−**0.069; 0.126)	0.57	**−**0.004	**−**0.001 (**−**0.104; 0.101)	0.98
Start ≤ 10 weeks	**−**0.075	0.088 (0.011; 0.166)	0.03	0.028	0.031 (**−**0.054; 0.115)	0.48
Preconceptional start	0.058	0.221 (0.147; 0.296)	<0.001	0.123	0.126 (0.037; 0.215)	<0.01

		Head circumference growth (SDS)				
		Basic^b^			Adjusted for covariates^c^	
		B^g^ (95 % CI)	*P*		B^g^ (95 % CI))	*P*
Period of head growth and folic acid supplementation						
Second and third trimester (*n* = 5832 (99.4 %), 11,323 observations)						
No use^f^		Reference			Reference	
Start > 10 weeks		0.002 (**−**0.008; 0.012)	0.72		0.001 (**−**0.009; 0.011)	0.84
Start ≤ 10 weeks		0.004 (**−**0.004; 0.012)	0.31		0.002 (**−**0.007; 0.010)	0.69
Preconceptional start		0.008 (**−**0.000; 0.016)	0.05		0.002 (**−**0.007; 0.012)	0.65
Birth through 6 years (*n* = 5515 (94.0 %), 29,976 observations)						
No use^f^		Reference			Reference	
Start > 10 weeks		**−**0.000 (**−**0.000; 0.000)	0.32		**−**0.000 (**−**0.000; 0.000)	0.26
Start ≤ 10 weeks		**−**0.000 (**−**0.000; 0.000)	0.45		**−**0.000 (**−**0.000; 0.000)	0.39
Preconceptional start		**−**0.000 (**−**0.000; 0.000)	0.15		**−**0.000 (**−**0.000; 0.000)	0.26

*SDS* standard deviation score

^a^Gender and gestational age-adjusted foetal and child head circumference standard deviation scores

^b^Model 1: no adjustments

^c^Model 2: model 1, additionally adjusted for maternal age, ethnicity, education, income, parity, BMI, psychopathology, smoking and alcohol consumption during pregnancy and the interactions between gestational age and maternal smoking, ethnicity and education

^d^Values represent offspring head size in SDS at 20 weeks of gestation (intercept) per group of folic acid supplementation

^e^Values represent Β (95 % CI) for the difference in offspring head size (intercept) for each group of folic acid supplementation compared with the reference group of ‘no use’ from mixed model regression analyses

^f^Reference group of primiparae women who never smoked or drank alcohol during pregnancy, with mean age of 29.8 years and mean BMI of 24.5 at enrolment, Dutch national origin, higher education, family income > € 2000/month and median Global Severity Index score of 0.17 in mid-pregnancy

^g^Values represent Β (95 % CI) for the interaction between maternal folic acid supplementation and gestational age (in weeks) from mixed model regression analyses, i.e. the difference in offspring head circumference growth (in SDS) per week for each group of folic acid supplementation compared with the reference group of ‘no use’

We found no evidence for an association between maternal folic acid supplementation during pregnancy and prenatal or postnatal head *growth* in the offspring (Table 2).

With regard to potential effect modification, none of the interaction terms of maternal folic acid use with child gender (either in pre- or postnatal models) were statistically significant (all *P* values > 0.10). Therefore, analyses were not further stratified by gender.

The effect of supplement use could not be attributed to other nutrients in the supplement: foetuses of mothers who started using any supplement before conception had a larger HC *size* at 20 weeks of gestation than those whose mother did not use supplements (fully adjusted models: B_‘preconceptional start folic acid’ vs ‘no use’_ = 0.131, 95 % CI 0.031; 0.232, *P* = 0.01; *B*_‘preconceptional start multivitamins’ vs ‘no use’_ = 0.115, 95 % CI 0.009; 0.221, *P* = 0.03). After additional adjustment for EFW, the latter association of maternal preconceptional multivitamin use with foetal HC size disappeared (*B* = 0.097, 95 % CI −0.005; 0.198, *P* = 0.06), whereas the association of preconceptional folic acid use with foetal HC size did not (*B* = 0.120, 95 % CI 0.025; 0.216, *P* = 0.01).

### Maternal folate concentrations and offspring head circumference

The first part of Table 3 shows the analyses of the associations of maternal folate concentrations with offspring head *size*. At 20 weeks of gestation, neither folate SD scores nor folate deficiency was associated with head size.

**Table 3:** Maternal folate concentration^a^/deficiency^b^ during pregnancy and offspring head size at 20 weeks of gestation, prenatal head growth and postnatal head growth^c^

	Head circumference size (SDS)					
	Basic^d^			Adjusted for covariates^e^		
	SDS^f^	B^g^ (95 % CI)	*P*	SDS^f^	B^g^ (95 % CI)	*P*
Head size (intercept (SDS) at 20 weeks gestation) and folate concentration (*n* = 5832 (99.4 %), 11,323 observations)						
Mean folate^h,i^	**−**0.055	Reference		0.074	Reference	
Folate (per SD)^j^	**−**0.021	0.034 (0.007; 0.061)	0.01	0.081	0.007 (**−**0.022; 0.037)	0.63
Normal folate^i^	**−**0.051	Reference		0.077	Reference	
Folate-deficient	**−**0.100	**−**0.049 (**−**0.148; 0.049)	0.32	0.110	0.033 (**−**0.068; 0.134)	0.52

		Head circumference growth (SDS)				
		Basic^d^			Adjusted for covariates^e^	
		B^k^ (95 % CI)	*P*		B^k^ (95 % CI)	*P*
Period of head growth and folate concentration						
Second and third trimester (*n* = 5832 (99.4 %), 11,323 observations)						
Folate (per SD)^h,i^		0.006 (0.003; 0.009)	<0.001		0.004 (0.001; 0.007)	0.01
Folate-deficient		**−**0.013 (**−**0.023; **−**0.002)	0.02		**−**0.007 (**−**0.018; 0.004)	0.19
Birth through 6 years (*n* = 5515 (94.0 %), 29,976 observations)						
Folate (per SD)^h,i^		**−**0.000 (**−**0.000; 0.000)	0.28		**−**0.000 (**−**0.000; 0.000)	0.66
Folate-deficient		0.000 (**−**0.000; 0.001)	0.06		0.000 (**−**0.000; 0.000)	0.29

*SDS* standard deviation score

^a^Folate concentration in standard deviation scores

^b^Folate deficiency was defined as a folate concentration < 7 nmol/L. Subjects without folate deficiency comprised the reference group ‘normal folate’

^c^Gender and gestational age-adjusted foetal and child head circumference standard deviation scores

^d^Model 1: adjusted for gestational age at venipuncture

^e^Model 2: model 1, additionally adjusted for maternal age, ethnicity, education, income, parity, BMI, psychopathology, smoking and alcohol consumption during pregnancy and the interactions between gestational age and: maternal smoking, ethnicity and education

^f^Values represent offspring head size in SDS at 20 weeks of gestation (intercept) per type of folate concentration from mixed model regression analyses

^g^Values represent Β (95 % CI) for the difference in offspring head size (intercept) for each type of folate concentration compared with its reference (‘mean folate’ or ‘normal folate’) from mixed model regression analyses

^h^Mean folate concentration: 0 SDS ≡ 17.4 nmol/L

^i^Reference group of primiparae women who never smoked or drank alcohol during pregnancy, with mean age of 29.8 years and mean BMI of 24.5 at enrolment, Dutch national origin, higher education, family income > € 2000/month and median Global Severity Index score of 0.17 in mid-pregnancy

^j^Standard deviation folate concentration: 8.8 nmol/L

^k^Values represent Β (95 % CI) for the interaction between maternal folate concentration/deficiency and gestational age (in weeks) from mixed model regression analyses, i.e. the difference in head circumference growth (in SDS) per week for each SD increase in folate concentration/between children of mothers with and without folate deficiency

In the basic linear regression models, higher maternal folate concentration was associated with a larger child head size at 20 and 30 weeks of gestation, at birth, at age 5–10 months and at 6 years (see Online Resource 1; note: size here in mm). Only at 30 weeks of gestation, this association was not accounted for by confounders. Additional adjustment for EFW attenuated the effect estimate, but the association remained statistically significant (*B* = 0.27, 95 % CI 0.03; 0.51, *P* = 0.03). Including quadratic terms of folate to the model did not improve the fit (data not shown). When using folate as a dichotomous variable, no associations were found between folate deficiency and offspring head size. Similar results were found using 8 nmol/L to define low maternal folate status.

The associations of maternal folate concentrations with offspring head *growth* are presented in Table 3. Higher maternal folate concentrations were associated with increased foetal head growth, and folate deficiency was associated with decreased foetal head growth in the basic models. These associations were attenuated when fully adjusted for confounders (main confounders: maternal ethnic background, income, BMI at enrolment and smoking during pregnancy). However, foetuses exposed to higher maternal folate concentrations in early pregnancy (continuous measure) showed increased head growth between the second and third trimester of pregnancy: for each 1-SD higher folate concentration, foetal head growth was 0.004 SD per week faster, even after additional adjustment for EFW (95 % CI 0.000; 0.007, *P* = 0.02). This results in an average estimated increase of 0.16 SD at birth, which translates into approximately 2.7 mm difference in HC at birth.

Again, including quadratic terms of folate to the model did not improve the fit (data not shown). In secondary analyses, maternal folate concentration was divided into quintiles, using the lowest quintile as the reference category (quintile 1). In a dose–response manner, foetuses of mothers with a folate concentration in the second through highest quintile had significantly greater prenatal head growth compared to foetuses of mothers with a folate concentration in the lowest quintile, although this difference was only statistically significant for those children of mothers with a folate concentration in the highest quintile (*B* = 0.012, *P* = 0.018, see Online Resource 2; *P* for trend = 0.018). However, we did not observe greater foetal head growth in foetuses of mothers with a folate concentration in the extreme high ends of the folate distribution (*B*_highest 10 % folate_ = 0.002, *P* = 0.64; *B*_highest 5 % folate_ = 0.003, *P* = 0.69) compared with foetuses of mothers with a folate concentration in the remainder of the distribution.

No associations were found between maternal folate concentrations or folate deficiency and postnatal head growth. These results did not change after exclusion of the birth measure of HC, which can be highly imprecise due to the moulding that takes place during the birth process. Again, none of the interaction terms of maternal folate concentration with child gender (either in pre- or postnatal models) were statistically significant (all *P* values > 0.10), and analyses were not further stratified by gender.

## Discussion

In this population-based study, we found small effects of maternal folic acid supplementation and folate concentration on head growth in the offspring. Maternal preconceptional start of folic acid supplementation was associated with larger prenatal head *size* at 20 weeks of gestation, but not with prenatal head *growth* after week 20 or postnatal head *growth*. Maternal folate concentrations—serving as nutritional biomarkers—were related to a faster foetal head growth from the second to the third trimester of pregnancy, resulting in a larger head size at 30 weeks of gestation. The associations were independent of overall foetal growth. Although the direction of the associations between very low or high folate status and foetal head growth was in line with those from analyses with the continuous measure of folate, our results suggest a linear effect of folate on HC rather than extremely low or high folate concentrations driving the association. No effect of maternal folate on HC was observed after birth.

### Folic acid supplementation versus folate concentrations

Folic acid supplement use and folate concentrations are correlated, but their correlation is moderate; the quantitative relationship between supplement and biomarker is influenced by various physiological and environmental factors [32]. Folate concentrations are more directly related to the biological processes in the human body than self-reported folic acid use. As such, they are a more precise measure of actual folate status, allowing for better detection of potential subtle effects. This may explain why maternal folate concentrations were associated with offspring head growth, whereas folic acid supplementation was not. Alternatively, in contrast to the categorical approach of folic acid supplementation, a continuous measure of folate concentration has statistically more power to detect differences.

In addition, although we measured folic acid supplementation retrospectively early in pregnancy, it covers the preconceptional period, whereas folate concentrations were measured around 13 weeks of gestation. Our measurements of HC started at 20 weeks of gestation. Since foetuses of mothers who preconceptionally started folic acid supplementation had a larger HC at 20 weeks of gestation compared to those whose mothers did not use folic acid, the effect of supplementation may already have occurred before 20 weeks of gestation.

### Prenatal versus postnatal head growth

We observed an association between maternal folate concentrations and offspring head growth prenatally, but not postnatally. Schlotz et al. [33] reported an association between maternal folate concentration measured at the same gestational age as in the current study (13.5 weeks) and HC at birth, but not 9 months after birth, concluding that maternal folate specifically affects prenatal head growth. Our findings of specific effects of maternal folate on foetal head growth, independent of overall growth, support and extend this conclusion. A plausible explanation may be the relatively close proximity of foetal HC measures to the measure of folate concentrations early in pregnancy, in contrast to the more distant measures of child HC. Additionally, small prenatal effects on head size may be compensated in postnatal life, when other factors can modulate neurodevelopment and brain plasticity, such as child nutrition and enriched environments. Further, animal studies have shown that folate can affect both embryonic and foetal brain development at various stages, including cell proliferation and differentiation, through epigenomic effects [34]. While the effect sizes we observed are marginal, small effects on head growth during prenatal life may have, for example, an exponential effect on the number of synaptic connections, which can influence the overall connectivity and plasticity potential of the developing brain. Each cortical neuron has on average between 1000 and 10,000 synapses. Thus, a small reduction in neurons can have a very large effect on the number of connections within the brain. This in turn may predispose the child to cognitive and behavioural impairment later in life. Such subtle changes in brain development, however, cannot be accurately measured postnatally with a simple proxy like HC. To improve measurement accuracy, alternative measures, such as brain MRI, will need to be used.

### Residual confounding

Residual confounding can never be excluded as an explanation for observed associations in epidemiological studies. Lifestyle and socio-economic factors are strongly related to folic acid supplement use, but also affect overall dietary intake and health status. This is reflected in the current study by the fact that folate-deficient women differed on many of these factors from women with a plasma folate concentration in the normal range. A decline in the effect of maternal folate concentration on foetal HC size and growth was noticeable after adjustment for confounders. Biomarkers providing information about supplement use can be confounded although they do not rely on self-report data and are thus less prone to information bias. Further (residual) confounding, for example in terms of other nutrients in the diet, cannot be excluded.

### Strengths and limitations

The strengths of our study are its large sample size, availability of plasma folate concentrations as opposed to dietary intake of folate, having multiple measures of offspring HC (starting in pregnancy) and the ability to adjust for a considerable number of covariates.

The study also has its limitations. First, our assessment of maternal folate status was based on a single measure in blood plasma. One measurement, although indicative, is not a reliable reflection of a mother’s long-term folate status. Additionally, red blood cell instead of plasma folate would be a better measure of long-term folate status. On the other hand, plasma folate can be more reliably measured than red blood cell folate, as the serum/plasma folate assay is less complex to perform and requires less steps in handling the sample before analysis than the red cell folate assay [35, 36]. To identify the most folate-sensitive window of foetal head growth, repeated measures of folate concentrations throughout pregnancy are needed. Further, ultrasound measures provide a global measure of brain growth and development and do not have the contrast to determine differences in cortical and subcortical development, development of the cortical sheet, or myelination. Prenatal MRI measurements could identify whether growth differences are global or involve specific brain regions.

### Relevance

Higher maternal folate concentrations were associated with slightly increased foetal head growth from the second to the third trimester. Additionally, preconceptional start of folic acid supplementation was associated with a larger head size at 20 weeks of gestation. In the Netherlands, only about half of the women use folic acid supplements during the conceptional period [18, 37, 38], and median dietary folate equivalent intake from foods and supplements ranges from 249 to 282 µg/day for women in reproductive age. Within the same population, median dietary folate equivalent intake from foods only ranges from 216 to 242 µg/day [39]. Whereas those women not using folic acid supplements in the conceptional period may consume enough folate from foods in order to prevent folate deficiency, they do not meet the recommended daily intake of 400 µg of folate. Although folic acid fortification could improve folate status, it would not provide the full requirement. It thus remains important to better educate women of childbearing age about the benefits of folic acid supplementation. Additionally, more research is needed to identify (1) the most folate-sensitive period of foetal head/brain growth, (2) whether specific brain regions are affected and (3) whether the effects of folate on foetal head growth influence children’s long-term functioning.

## Electronic supplementary material

Below is the link to the electronic supplementary material.

Supplementary material 1 (DOC 72 kb)

Supplementary material 2 (DOC 38 kb)
