# Supplementary material for: Associations of maternal folic acid supplementation and folate concentrations during pregnancy with foetal and child head growth: the Generation R Study
Source: Eur J Nutr. 2015 Oct 26;56(1):65–75. doi: 10.1007/s00394-015-1058-z (PMC5290045; doi:10.1007/s00394-015-1058-z)
Supplement: Supplementary file 1 — Supplementary material 1 (DOC 72 kb) [file 394_2015_1058_MOESM1_ESM.doc]

**European Journal of Nutrition**

**Associations of maternal folic acid supplementation and folate concentrations during pregnancy with foetal and child head growth. The Generation R Study**

Jolien Steenweg-de Graaff, PhD, Sabine J Roza, MD, PhD, Alette N Walstra, MD, Hanan El Marroun, PhD, Eric AP Steegers, MD, PhD, Vincent WV Jaddoe, MD, PhD, Albert Hofman, MD, PhD, Frank C Verhulst, MD, PhD, Henning Tiemeier, MD, PhD, and Tonya White MD, PhD

**Corresponding Author:**

Tonya White, MD, PhD

Department of Child and Adolescent Psychiatry

Erasmus Medical Centre Rotterdam-Sophia Children's Hospital

P.O. Box 2060

3000 CB Rotterdam

The Netherlands

Phone: +31 10 7037072

Fax: +31 10 7032111

E-mail: [t.white@erasmusmc.nl](mailto:t.white@erasmusmc.nl)

| **Online resource 1. Maternal folate concentration^a^/deficiency^b^ during pregnancy and foetal/child head circumference size^c^** | | | | | | |
| --- | --- | --- | --- | --- | --- | --- |
|  | Head circumference size (mm) | | | | | |
|  | Basic^d^ | | Adjusted for covariates^e^ | | | |
|  | B (95% CI) | *P* | B (95% CI) | | *P* | |
| Time of HC measurement and folate concentration |  |  |  | |  | |
| 20 weeks (n=5670, 96.7%) |  |  |  | |  | |
| Folate (per SD) | 0.20 (0.04;0.36) | 0.02 | 0.00 (-0.18;0.18) | 0.99 | |  |
| Folate-deficient | -0.37 (-0.94;0.21) | 0.21 | 0.22 (-0.38;0.82) | 0.47 | |  |
|  |  |  |  | |  |  |
| 30 weeks (n=5653, 96.4%) |  |  |  | |  |  |
| Folate (per SD) | 0.94 (0.70;1.18) | <0.001 | 0.47 (0.21;0.74) | | <0.001 |  |
| Folate-deficient | -1.84 (-2.72;-0.97) | <0.001 | -0.47 (-1.36;0.41) | | 0.30 |  |
|  |  |  |  | |  |  |
| birth (n=3226, 55.0%) |  |  |  | |  |  |
| Folate (per SD) | 0.62 (0.06;1.18) | 0.03 | 0.19 (-0.42;0.81) | | 0.53 |  |
| Folate-deficient | -2.64 (-4.71;-0.58) | 0.01 | -1.41 (-3.51;0.69) | | 0.19 |  |
|  |  |  |  | |  |  |
| 3 months (n=3287, 56.0%) |  |  |  | |  |  |
| Folate (per SD) | 0.16 (-0.23;0.54) | 0.42 | 0.09 (-0.32;0.51) | | 0.66 |  |
| Folate-deficient | -0.95 (-2.43;0.53) | 0.21 | -0.53 (-2.05;0.99) | | 0.50 |  |
|  |  |  |  | |  |  |
| 5-10 months (n=4132, 70.4%) |  |  |  | |  |  |
| Folate (per SD) | 0.47 (0.11;0.84) | 0.01 | 0.37 (-0.03;0.78) | | 0.07 |  |
| Folate-deficient | -1.48 (-2.91;-0.06) | 0.04 | -0.83 (-2.31;0.65) | | 0.27 |  |
|  |  |  |  | |  |  |
| 6 years (n=4036, 68.8%) |  |  |  | |  |  |
| Folate (per SD) | 0.69 (0.20;1.17) | <0.01 | 0.33 (-0.20;0.85) | | 0.22 |  |
| Folate-deficient | **-**0.81 (**-**2.69;1.07) | 0.399 | 0.18 (**-**1.76;2.11) | | 0.86 |  |
| Values at the 20 week head circumference (HC) measurement differ from those reported in Table 3, first part: size here in mm instead of standard deviation score (SDS)  ^a^ Folate concentration in standard deviation scores, mean folate concentration: 0 SDS ≡ 17.4 nmol/L, 1 SD = 8.8 nmol/L.  ^b^ Folate deficiency was defined as a folate concentration < 7 nmol/L. Subjects without folate deficiency comprised the reference group ‘normal folate’.  ^c^ Values represent Β (95% CI) for the difference in offspring head size for each type of folate concentration compared with its reference (‘mean folate’ or ‘normal folate’) from linear regression analyses, head circumference in mm.  ^d^ Model 1: adjusted for gestational age at venipuncture, gender and gestational age of the foetus/child.  ^e^ Model 2: model 1, additionally adjusted for maternal age, ethnicity, education, income, parity, BMI, and psychopathology, smoking and alcohol consumption during pregnancy. | | | | | |  |
