# Supplementary material for: Associations of maternal folic acid supplementation and folate concentrations during pregnancy with foetal and child head growth: the Generation R Study
Source: Eur J Nutr. 2015 Oct 26;56(1):65–75. doi: 10.1007/s00394-015-1058-z (PMC5290045; doi:10.1007/s00394-015-1058-z)
Supplement: Supplementary file 2 — Supplementary material 2 (DOC 38 kb) [file 394_2015_1058_MOESM2_ESM.doc]

**European Journal of Nutrition**

**Associations of maternal folic acid supplementation and folate concentrations during pregnancy with foetal and child head growth. The Generation R Study**

Jolien Steenweg-de Graaff, PhD, Sabine J Roza, MD, PhD, Alette N Walstra, MD, Hanan El Marroun, PhD, Eric AP Steegers, MD, PhD, Vincent WV Jaddoe, MD, PhD, Albert Hofman, MD, PhD, Frank C Verhulst, MD, PhD, Henning Tiemeier, MD, PhD, and Tonya White MD, PhD

**Corresponding Author:**

Tonya White, MD, PhD

Department of Child and Adolescent Psychiatry

Erasmus Medical Centre Rotterdam-Sophia Children's Hospital

P.O. Box 2060

3000 CB Rotterdam

The Netherlands

Phone: +31 10 7037072

Fax: +31 10 7032111

E-mail: [t.white@erasmusmc.nl](mailto:t.white@erasmusmc.nl)

| **Online Resource 2. Maternal folate concentration during pregnancy by quintiles and offspring prenatal head growth (n=5832 (99.4%), 11323 observations).^a^** | | | | |
| --- | --- | --- | --- | --- |
|  | Head circumference growth (SDS) | | | |
|  | Basic^b^ | | Adjusted for covariates^c^ | |
|  | B^d^ (95% CI) | *P* | B^d^ (95% CI) | *P* |
| Maternal folate^e^ |  |  |  |  |
| Quintiles (folate) |  |  |  |  |
| 1 (<= 9.00) | Reference |  | Reference |  |
| 2 (9.01-13.00) | 0.007 (-0.002;0.016) | 0.96 | 0.001 (-0.008;0.010) | 0.86 |
| 3 (13.01-18.70) | 0.010 (0.008;0.019) | 0.09 | 0.001 (-0.008;0.010) | 0.81 |
| 4 (18.71-25.70) | 0.011 (0.002;0.020) | 0.035 | 0.003 (-0.006;0.013) | 0.51 |
| 5 (> 25.70) | 0.020 (0.011;0.029) | 0.014 | 0.012 (0.002;0.021) | 0.018 |
| *P* for trend |  | <0.001 |  | 0.018 |
| *SDS* standard deviation score  ^a^ Gender and gestational age adjusted foetal head circumference standard deviation scores  ^b^ Model 1: adjusted for gestational age at venipuncture.  ^c^ Model 2: model 1, additionally adjusted for maternal age, ethnicity, education, income, parity, BMI, and psychopathology, smoking and alcohol consumption during pregnancy and the interactions between gestational age and: maternal smoking, ethnicity, and education.  ^d^ Values represent Β (95% CI) for the interaction between maternal folate status and gestational age (in weeks) from mixed model regression analyses, i.e. the difference in head circumference growth (in SDS) per week between children of mothers with a folate concentration in the second through highest quintile of the distribution and children of mothers with a folate concentration in the lowest quintile of the distribution.  ^e^ Folate in nmol/L. | | | | |
